# AN ACADEMIC-HEALTH SYSTEM COLLABORATION TO DEVELOP A PROGRAM FOR FAMILY CAREGIVERS OF PERSONS WITH DEMENTIA

**DOI:** 10.1093/geroni/igz038.1647

**Published:** 2019-11-08

**Authors:** Elena Siegel, Ladson Hinton, Elizabeth Rice, Nilpa Shah, Vanessa McElroy, Jasmine DeGuzman Lacsamana, Heather M Young, Gregory A Maynard

**Affiliations:** 1 Betty Irene Moore School Of Nursing, University Of California Davis, Sacramento, California, United States; 2 University of California Davis Health, Sacramento, California, United States; 3 Archstone Foundation, Long Beach, California, United States

## Abstract

Hospitalization of persons with dementia can pose specific challenges for family caregivers, with post-discharge issues in managing acute clinical care needs coupled with dementia-related care that can exacerbate caregiver fatigue and capacity. We established an academic-practice collaboration to develop an evidence-based and innovative multi-component health system-level program to support family caregivers of persons with dementia in transition from hospital to community. Using an implementation science approach aimed to decrease the gap in translation of caregiver research into practice, we co-designed the program/implementation plan as a quality improvement initiative reflecting an integration of evidence from family caregiving literature and the health system’s unique context, workflows, stakeholder perspectives, resources, and values/priorities. This paper highlights insights gained and lessons learned in establishing a successful academic-practice collaboration, including time/investment to establish a shared project vision and identify/leverage existing organizational capacity to successfully deliver a program to improve the health and wellbeing of family caregivers.

